# Suppression of *AMPK/aak-2* by NRF2/SKN-1 down-regulates autophagy during prolonged oxidative stress

**DOI:** 10.1096/fj.201800565RR

**Published:** 2018-10-02

**Authors:** Monika Kosztelnik, Anita Kurucz, Diana Papp, Emily Jones, Timea Sigmond, Janos Barna, Maria H. Traka, Tamas Lorincz, Andras Szarka, Gabor Banhegyi, Tibor Vellai, Tamas Korcsmaros, Orsolya Kapuy

**Affiliations:** * Department of Genetics, Eötvös Loránd University, Budapest, Hungary;; † Department of Medical Chemistry, Molecular Biology, and Pathobiochemistry, Semmelweis University, Budapest, Hungary;; # Pathobiochemistry Research Group, Hungarian Academy of Sciences, Semmelweis University, Budapest, Hungary;; ‡ Gut Health and Microbes, Quadram Institute, Norwich Research Park, Norwich, United Kingdom;; § Earlham Institute, Norwich, United Kingdom;; ¶ Food Innovation and Health, Quadram Institute, Norwich, United Kingdom;; ‖ Laboratory of Biochemistry and Molecular Biology, Department of Applied Biotechnology and Food Science, Budapest University of Technology and Economics, Budapest, Hungary;

**Keywords:** AMPK, prolonged stress, aging, *C. elegans*, network

## Abstract

NF-E2–related factor 2 (NRF2) transcription factor has a fundamental role in cell homeostasis maintenance as one of the master regulators of oxidative and electrophilic stress responses. Previous studies have shown that a regulatory connection exists between NRF2 and autophagy during reactive oxygen species–generated oxidative stress. The aim of the present study was to investigate how autophagy is turned off during prolonged oxidative stress, to avoid overeating and destruction of essential cellular components. AMPK is a key cellular energy sensor highly conserved in eukaryotic organisms, and it has an essential role in autophagy activation at various stress events. Here the role of human AMPK and its *Caenorhabditis elegans* counterpart AAK-2 was explored upon oxidative stress. We investigated the regulatory connection between NRF2 and AMPK during oxidative stress induced by *tert*-butyl hydroperoxide (TBHP) in HEK293T cells and *C. elegans*. Putative conserved NRF2/protein skinhead-1 binding sites were found in *AMPK/aak-2* genes by *in silico* analysis and were later confirmed experimentally by using EMSA. After addition of TBHP, NRF2 and AMPK showed a quick activation; AMPK was later down-regulated, however, while NRF2 level remained high. Autophagosome formation and Unc-51–like autophagy activating kinase 1 phosphorylation were initially stimulated, but they returned to basal values after 4 h of TBHP treatment. The silencing of NRF2 resulted in a constant activation of AMPK leading to hyperactivation of autophagy during oxidative stress. We observed the same effects in *C. elegans* demonstrating the conservation of this self-defense mechanism to save cells from hyperactivated autophagy upon prolonged oxidative stress. We conclude that NRF2 negatively regulates autophagy through delayed down-regulation of the expression of AMPK upon prolonged oxidative stress. This regulatory connection between NRF2 and AMPK may have an important role in understanding how autophagy is regulated in chronic human morbidities characterized by oxidative stress, such as neurodegenerative diseases, certain cancer types, and in metabolic diseases.—Kosztelnik, M., Kurucz, A., Papp, D., Jones, E., Sigmond, T., Barna, J., Traka, M. H., Lorincz, T., Szarka, A., Banhegyi, G., Vellai, T., Korcsmaros, T., Kapuy, O. Suppression of *AMPK/aak-2* by NRF2/SKN-1 down-regulates autophagy during prolonged oxidative stress.

Oxidative stress is an inevitable and often disadvantageous condition affecting essentially all living organisms. It can arise from the imbalance of the antioxidant defense mechanisms and the reactive oxygen species (ROS) such as hydroxyl ^•^OH, superoxide O_2_^•–^, and hydrogen peroxide H_2_O_2_, and reactive nitrogen species such as nitric monoxide ^•^NO (1). These highly reactive molecules can be produced endogenously (*e.g.*, in mitochondria, peroxisome, cytosol) or generated due to exposure to numerous exogenous agents (*e.g.*, infrared, UV, cytokines, growth factors, drugs, toxins, high temperature) (2). By taking electrons from macromolecules, ROS can generate harmful chemical processes leading to alteration and damage of cell structure, or even to cell death (3). If oxidative stress–induced defense mechanisms are not sufficient, or the stress is prolonged, it leads to many pathophysiologic conditions (atherosclerosis, cancer, diabetes, cardiovascular diseases, chronic inflammation, stroke, and neurodegenerative diseases such as Parkinson’s or Alzheimer’s disease) (4). If cells cannot recover by eliminating the harmful effects of oxidative stress, programmed cell death (*i.e.*, apoptosis, necrosis, autophagic cell death) can occur to maintain the functional integrity of the affected tissue (1).

The major transcriptional regulator of the antioxidant stress response in animals is NF-E2–related factor 2 [NRF2 (NFE2L2)] (5, 6). NRF2 regulates the cellular antioxidant response mechanism by modulating the expression of hundreds of cytoprotective genes with antioxidant response elements (AREs) in their promoters, including antioxidant enzymes, phase II detoxifying enzymes, xenobiotic transporters, and other stress response proteins (5, 7). At physiologic conditions, NRF2 has some basal level, but it is kept inactive by binding to a stoichiometric inhibitor labeled Kelch-like ECH-associated protein 1 (KEAP1) and targeted for ubiquitination (8, 9). Various oxidative agents result in conformational changes of KEAP1, thereby blocking NRF2 degradation. In *Caenorhabditis elegans*, the NRF2 protein is represented by the 3 currently known isoforms of the protein skinhead-1 (SKN-1). Although NRF2 works as a dimer, SKN-1 binds to the DNA in a unique monomeric form (5, 10). SKN-1 functions similarly to NRF2 in response to oxidative stress and is required for oxidative stress resistance and longevity in *C. elegans*. NRF2 also up-regulates other processes, including a cellular self-digesting pathway called autophagy (11). During oxidative stress, autophagy is a key element of cellular homeostasis that eliminates harmful agents from the cytosol such as protein aggregates and damaged mitochondria, and it thereby limits ROS production and restores oxidative balance. It also has an important role in guaranteeing the proper functions of the cell by self-digesting the dispensable elements of the cell during starvation. The focus of the present study was on the regulation of the most well-known type of autophagy, namely (macro)autophagy (12).

The conjugated form of the microtubule-associated protein 1A/1B-light chain 3 (LC3-II) is recruited to autophagosomal membranes, and the LC3-II level thus directly estimates the abundance of autophagosomes before lysosomal digestion. Because it reflects the autophagic activity, detecting LC3-II levels is a reliable and well-characterized method for monitoring autophagy flux and autophagy-related processes (13). The p62 protein binds both LC3 and unfavorable proteins; therefore, p62-targeted proteins are selectively transferred to the autophagosome and can be removed by autophagy (14, 15). In the subsequent degradation, both p62 and its substrate are destroyed; consequently, the p62 level quickly drops during intensive autophagy (16, 17). Blocking of autophagy results in an increase in cellular p62 level, and this feature is therefore used to detect dysfunctional autophagy (18, 19).

The induction of autophagosome formation is controlled by the Unc-51–like autophagy activating kinase 1 (ULK1) complex, containing ULK1, ATG13, and FIP200 (20). ULK1 activity is tightly regulated by the nutrient and energy sensors of cellular homeostasis, mammalian target of rapamycin (mTOR), and AMPK, respectively (21). Because mTOR is fully active at physiologic conditions to maintain general protein synthesis in the cell, mTOR is a key negative regulator of the initial steps of autophagy (22). However, AMPK becomes activated when the ATP:ADP ratio is dropping, and it induces autophagy. AMPK activation in turn inhibits mTOR, and AMPK can also induce ULK1 activity directly (through phosphorylation of Ser317, Ser555, and Ser777) (21, 23, 24).

The most relevant connection of autophagy and oxidative stress response mechanism is achieved *via* NRF2. Namely, activation of NRF2 occurs by the NRF2-dependent transcription of the ubiquitin-binding and autophagosome cargo protein p62. p62 quickly binds the NRF2 inhibitor (KEAP1) upon oxidative stress, resulting in the fast activation of the transcription factor (25). Recently, active NRF2 was found to bind to the ARE sequence of various autophagy regulators (*e.g*., *Sqstm1/p62*, *ATG4*, *ATG5*, *ATG7*, *Gabarapl1*), suggesting that NRF2 has a positive effect on autophagy (26). In addition, AMPK can also stimulate NRF2 signaling upon oxidative stress (21, 27). AMPK directly phosphorylates NRF2 at Ser550, promoting nuclear accumulation of NRF2 for ARE-mediated gene transcription (28). AMPK also induces the antioxidative heme oxygenase (*HO-1*) gene expression in human vascular cells and rat arteries *via* the NRF2/ARE pathway (29). In *C. elegans*, metformin activates AAK-2 (the worm ortholog of AMPK) to promote SKN-1/NRF2 nuclear translocation (30). Although several papers have shown the importance of autophagy activation upon oxidative stress, often *via* NRF2 (reviewed in refs. 31–33), how autophagy is down-regulated when oxidative stress is already balanced to avoid overeating and destruction of further and essential cellular components has not yet been explored.

Previously, genetic studies found that autophagy down-regulation could indeed be beneficial upon prolonged oxidative stress, and, interestingly, this survival mechanism involves NRF2. Anthocyanins from Chinese bayberry extract, while activating NRF2 in β cells, were found to negatively regulate oxidative stress–induced autophagy, and this outcome provided a protective effect after the transplantation of rat β cells under the renal capsules of the recipient mice (34). In another study, NRF2 was induced by an anticancer redox agent, mitoquinone, and the direct involvement of NRF2 in autophagy down-regulation was confirmed with genetic studies (35). Similarly, in mice, *NRF2* deficiency contributed to autophagy deregulation upon age-related retinopathy, implying the importance of NRF2 regulation of autophagy upon prolonged oxidative stress (36). In cardiomyocytes exposed to prolonged oxidative stress, AMPK levels were found to be decreased, resulting in autophagy inhibition (37). Down-regulation of autophagy in these studies was not detected when NRF2 activity was compromised, indicating a direct role for NRF2 in autophagy inhibition. Importantly, autophagy down-regulation in these studies was not observed when oxidative stress was short term, explaining why most of the previous oxidative stress–related experiments focusing on NRF2 and autophagy could not find this negative regulatory effect. To our knowledge, none of the published works provided a molecular mechanism for how NRF2 could down-regulate autophagy. Identifying which genes and how they are involved in the adaptation to prolonged oxidative stress will contribute to our understanding of antioxidant stress response and the pathogenesis of related diseases, including cardiovascular disease and cancer, as well as aging.

Prompted by these questions, in the present study we investigated the relationship between NRF2 and autophagy. Using computational regulatory network analysis, we identified a conserved NRF2 binding site in the promoter region of AMPK. We validated the functional role of this binding site, and also the critical effect of NRF2 in autophagy down-regulation, both in human cell lines and in the model organism *C. elegans*. Our results uncovered the mechanism of how NRF2 inhibits autophagy hyperactivation upon prolonged oxidative stress.

## MATERIALS AND METHODS

### Cell culture and oxidative treatment

Human embryonic kidney (HEK293T) and human colonic (Caco-2) cells were cultured in DMEM (41965039; Thermo Fisher Scientific, Waltham, MA, USA) supplemented with 10% fetal bovine serum (10500064; Thermo Fisher Scientific) and 1% antibiotics/antimycotics (15240062; Thermo Fisher Scientific). This cell line was maintained in adherent culture in a humidified incubator at 37°C in 95% air and 5% CO_2_. To simulate oxidative stress, LuperosTBH70x, *tert*-butyl hydroperoxide (TBHP) solution (458139; MilliporeSigma, Burlington, MA, USA) was used for different lengths of time (*i.e.*, 0.5, 1, 2, and 3 h).

### Immunofluorescence

HEK293T cells (7 × 10^4^) were seeded on glass coverslips (ECN 631–1577; VWR International, Radnor, PA, USA). Cells were washed in PBS, fixed with ice-cold 100% methanol for 10 min, and washed again. Coverslips were blocked for 30 min with PBS buffer with 0.1% Tween-20 containing 3% bovine serum albumin (A 9647; MilliporeSigma) and then incubated overnight at 4°C with primary antibody (LC3 A/B, 4108S; Cell Signaling Technology, Danvers, MA, USA) at 1:200 dilution in 1% bovine serum albumin containing PBS buffer with 0.1% Tween-20. Samples were washed with PBS and then incubated for 1 h at room temperature with Alexa Fluor 488 (4412S; Cell Signaling Technology) secondary antibody at 1:600 dilution. Cells were washed with PBS and treated with DAPI at 1:1000 dilution in PBS for 10 min, followed by wash with PBS. Coverslips were mounted by using FluorSave Reagent (345789; MilliporeSigma) and visualized by using a Zeiss LSM 710 laser confocal microscope (Carl Zeiss Microscopy GmbH, Jena, Germany). Three images per given conditions were taken. All data are expressed as means ± sem. Comparisons between groups were made by using ANOVA with Tukey’s multiple comparison *post hoc* test. Statistical significance was evaluated, and values of *P* < 0.05 were considered significant.

### Quantification of mRNA

Total RNA was isolated from HEK293T cells at 3 d of cultivation by using Trizol reagent (Thermo Fisher Scientific). RNA was reverse transcribed by using the SuperScript II First-Strand Synthesis System (Thermo Fisher Scientific). Quantification of *NRF2* and *AMPK* mRNA was performed by using the GoTaq Quantitative PCR (qPCR) Master Mix (A6001; Promega, Madison, WI, USA). Forward and reverse primer sequences used for qPCR were as follows: *NRF2*: 5′-TCCAGTCAGAAACCAGTGGAT-3′and 5′-GAATGTCTGCGCCAAAAGCTG-3′; *AMPK*: 5′-ACTGTACCAGGTCATCAGTACACC-3′and 5′-TCCAGGTACATCAGATTTCCTTC-3′; and *GAPDH*: 5′-TGCACCACCAACTGCTTAGC-3′ and 5′-GGCATGGACTGTGGTCATGAG-3′. The PCR thermal program included the following: 10 min at 95°C (1 cycle), and 30 s at 95°C, 45 s at 58°C, 30 s at 72°C (40 cycles), 5 min at 95°C, 1 min at 55°C, 30 s at 97°C (1 cycle). Measures were performed on an Mx3005P qPCR System (Stratagene, San Diego, CA, USA). Melting curve analysis was performed to confirm correct PCR product size and absence of nonspecific bands. Relative mRNA levels were determined by normalizing the PCR threshold cycle number of *NRF2* and *AMPK* with that of the *GAPDH* reference gene. Normalized data were analyzed by using independent 2-sample Student’s *t* tests with Bonferroni correction (SPSS v.20 software; IBM, Armonk, NY, USA).

### RNA interference in HEK293T cells

In the case of human cells, 1 d before the transfection, 2.5 × 10^5^ HEK293T cells were plated in 2.5 ml growth medium without antibiotics. Duplex RNA to targeting human NRF2 [sense 5′-GGUUGAGACUACCAUGGUU(dTT)-3′ antisense 5′-AACCAUGGUAGUCUCAACC(dTT)]-3′ was transfected into cells at 50–60% confluency. Small interfering RNA (siRNA) and Lipofectamine RNAiMax were diluted into Opti-MEM I (GlutaMax-I) in accordance with the manufacturer’s protocol (Thermo Fisher Scientific). siRNA duplex-Lipofectamine RNAiMax complexes were added to each well. Cells were assessed at 24 h after transfection by using real-time PCR. For negative control scramble, siRNA was used.

### Western blot analysis

Treated HEK293T or Caco-2 cells were collected and lysed by using RIPA buffer (20 mM Tris, 135 mM NaCl, 10% glycerol, 1% NP40, pH 6.8). Protein concentrations were measured by using Pierce BCA Protein Assay (Thermo Fisher Scientific, 23225). Equal amounts of protein were separated on a 10–15% SDS-PAGE gel and transferred to PVDF membrane (0.45; MilliporeSigma). Membranes were blocked with 5% nonfat dry milk or 1% bovine serum albumin in Tris-buffered saline, 0.1% Tween for 1 h followed by overnight incubation at 4°C with primary antibodies in 1% milk or 1% bovine serum albumin. The antibody sources are as follows: phospho-AMPKα Thr172 (2531S; Cell Signaling Technology), AMPKα (23A3; Cell Signaling Technology), NAD(P)H:quinone oxidoreductase 1 (NQO1) (A180; Cell Signaling Technology), anti-LC3β (sc-16755; Santa Cruz Biotechnology, Dallas, TX, USA), 4EBP1P (9459S; Cell Signaling Technology), phospho-ULK1 Ser555 (5869S; Cell Signaling Technology), ULK1 (8054S; Cell Signaling Technology), anti–glyceraldehyde-3-phosphate dehydrogenase (GAPDH) (sc-32233; Santa Cruz Biotechnology), HO-1 (D60G11; Cell Signaling Technology), NRF2 (C15410242; Diagenode, Denville, NJ, USA), and horseradish peroxidase conjugated secondary antibodies (sc-2020, sc-2005, Santa Cruz Biotechnology; 7074S, 7076S, Cell Signaling Technology). To detect both the phosphorylated and total forms of the same proteins, equal amounts of samples were blotted onto different PVDF membranes. Cross-reactivity and protein removal problems were avoided by using this approach. Each experiment was repeated 3 times. The quantification of Western blot bands was conducted on ImageQuant 5.2 software (GE Healthcare Life Sciences, Little Chalfont, United Kingdom). Relative band densities were normalized to appropriate total protein or GAPDH used as reference protein. The average of 3 independent measurements was calculated. All data are expressed as means ± sem. Comparisons between groups were made by using ANOVA with Tukey’s multiple comparison *post* *hoc* test. Statistical significance was evaluated, and values of *P* < 0.05 were considered significant.

### Measurement of oxidative stress by flow cytometry

HEK cells were seeded on 6-well plates and treated as indicated. After treatment, cells were trypsinized, washed once with PBS, and 0.5 × 106 cells/ml were labeled with 10 μM dichlorofluorescein-diacetate (DCF) or 2 μM Bodipy-[^11^C] (BOD) for 30 min in HBSS at 37°C. After labeling, cells were washed once with PBS, resuspended in PBS, and analyzed with a BD FACSCalibur flow cytometer (BD Biosciences, Franklin Lakes, NJ, USA). The fluorescence of DCF and BOD from excitation with a 488 nm laser was recorded in FL1 (530 nm). Cell population was gated through the SSC-FSC scatter plot by using unlabeled cells, and histograms normalized to the samples’ mode were constructed by using FlowJo software (FlowJo, Ashland, OR, USA). A minimum of 10,000 cells were analyzed per condition.

### Nuclear extracts and 2-color fluorescence EMSA

Nuclear extracts were prepared from Caco-2 cells treated with 100 µM TBHP for 4 h, using the Nuclear Extraction Kit (Abcam, Cambridge, MA, USA) according to the manufacturer’s instructions. Single-stranded oligonucleotide probes containing either AMPK query Nrf2 binding site (5′-AAACCATGACTCTGCATAAAA-3′ and 5′-GATCTTTTATGCNNNGTCAATG-3′) or AMPK NRF2 consensus binding site (5′-GATCTTTTATGCTGTGTCATGG-3′ and 5′-AAACCATGACACAGCATAAAA-3′) were manufactured by Integrated DNA Technologies (Coralville, IA, USA) and annealed at 95°C for 5 min, then cooled slowly to room temperature for several hours. Gel shift analysis was conducted by using the EMSA kit with SYBR Green and SYPRO Ruby (Thermo Fisher Scientific) according to the manufacturer’s instructions. Briefly, binding reactions were performed in a total volume of 10 μl containing 5 μg of nuclear extract, 1 ng of DNA probe, and 5 times binding buffer (750 mM KCl, 0.5 mM DTT, 0.5 mM EDTA, 50 mM Tris, pH 7.4) for 20 min at room temperature. In supershift experiments, nuclear extracts were preincubated with 2 μg of anti-Nrf2 antibody (sc365949-X; Santa Cruz Biotechnology) for 30 min before the addition of the oligonucleotide probe. At the end of the incubation period, 6× gel-loading solution was added to the binding reaction and protein–probe complexes separated by electrophoresis on a nondenaturing Novex 4–20% Tris-Glycine Mini Gel (Thermo Fisher Scientific) at 100 V for 2 h at 4°C. To visualize nucleic acids, the gel was stained with SYBR Green EMSA gel stain in TBE buffer (89 mM Tris base, 89 mM boric acid, 1 mM EDTA, pH ∼8.0), washed in dH_2_O, and imaged by using the AlphaImager system (ProteinSimple, San Jose, CA, USA). Proteins were subsequently visualized on the same gel by using SYPRO Ruby EMSA gel stain. After washing in dH_2_O, proteins were imaged by using the AlphaImager system. Gel bands were quantified by using the ImageJ/Fiji software package (National Institutes of Health, Bethesda, MD, USA).

### *C. elegans* strains and maintenance

Unless otherwise indicated, nematodes were maintained and propagated at 20°C on nematode growth medium (NGM)-containing plates and fed with *Escherichia coli* OP50 bacteria. Experiments were performed at 20°C on young adult hermaphrodites. The following *C. elegans* strains were used in this study: Bristol (N2) as wild-type, BU071 green flurescent protein (GFP)::LGG-1, TG38 *aak-2(gt33*), *atg-11(tm2508)*, EU31 *skn-1(zu135) IV/nT1 [unc-?(n754) let-?] (IV;V)* [*zu135* allele is a nonsense mutation that prevents DNA binding by all 3 SKN-1 isoforms (38)], *skn-1(lax120)*, TTV453 *aak-2::gfp;skn-1(zu135)*, GFP::LGG-1;*aak-2(gt33)*. The translational fusion *aak-2::gfp* reporter strain was a kind gift of Hyeon-Sook Koo (39).

### RNA interference treatment in *C. elegans*

*skn-1* was silenced by feeding *C. elegans* with *E. coli* HT115 bacteria that expressed *skn-1* double-stranded RNA from the vector pL4440. The *skn-1* RNA interference (RNAi) bacteria were a kind gift of T. Keith Blackwell (40). Overnight culture of *skn-1* RNAi bacteria were seeded onto NGM plates containing 50 μg/ml ampicillin, 6.25 μg/ml tetracycline, and 0.4 mM isopropyl β-d-thiogalactoside (R0393; Thermo Fisher Scientific) in final concentration. Worms were grown on RNAi bacteria from hatching until the L4/young adult stage. Empty vector containing HT115 bacteria was used as a control in the assays.

### Oxidative stress (paralysis) assay in *C. elegans*

Twenty to thirty age-synchronized young adult worms were transferred onto NGM or RNAi plates, respectively, containing 10 mM TBHP (A13926; Alfa Aesar, Ward Hill, MA, USA). All TBHP plates were prepared 1 d before the assays. Mobile and paralyzed animals were counted in every hour. Animals were counted as paralyzed if they failed to move upon prodding with a worm pick. At least 15 worms were assayed in all conditions, and the experiments were repeated 3 times. All experiments were conducted at 20°C. Statistical significance was determined by using independent 2-sample Student’s *t* tests.

### Fluorescent microscopy

For fluorescent microscopy, young adult worms were immobilized by using 0.1 M levamisole or 10 mM NaN_3_ in M9 buffer. Images for quantitative analysis were taken by using an Olympus BX51 microscope (Shinjuku, Tokyo, Japan), Leica DMI6000B microscope (Leica Camera AG, Wetzlar, Germany), or Zeiss AxioImager Z1. To observe AAK-2::GFP expression upon oxidative stress, age-synchronized L4 larvae were transferred to TBHP assay plates containing 1, 2, or 4 mM TBHP; NGM or RNAi plates without TBHP were used as control. Images of whole animals were taken after 1, 5, or 24 h incubation, and fluorescence intensity was measured by using ImageJ software. At least 15 worms were analyzed in all conditions, and the experiments were repeated at least 3 times. To study GFP::LGG-1 puncta upon oxidative stress, young adult age-synchronized worms were incubated on TBHP assay plates (1 mM TBHP) for 3 h. Epifluorescence images of the whole animals and the first 2 intestinal cells behind the pharynx were taken and analyzed by using ImageJ software; the GFP::LGG-1 positive area were measured relative to the area of the cell on each image. Up to 36 worms were analyzed in all conditions, and the experiments were repeated 2 times. All experiments were conducted at 20°C. Statistical significance was determined by using independent 2-sample Student’s *t* tests.

## RESULTS

### Putative conserved NRF2/SKN-1 binding sites were found in *AMPK/aak-2* genes

Previously, we developed an online resource, NRF2ome (*http://nrf2.elte.hu/*), which provides an integrated and systems-level database for NRF2 (6, 41). NRF2ome contains experimentally verified and predicted interactions of NRF2, and it lists known and potential NRF2 target genes, NRF2-regulating transcriptional factors, and microRNAs (41). We also developed the Autophagy Regulatory Network database (*http://arn.elte.hu/*) that contains autophagy components (proteins involved in the mechanisms of autophagy), their regulators, and their transcription factors (42). Comparing NRF2 target genes from NRF2ome with autophagy genes from the Autophagy Regulatory Network database, we found several autophagy-related genes that contain NRF2/ARE–binding sites (GCNNNGTCA) (43) in their promoter or coding region. We focused on the AMPK gene, because He *et al.* (37) had showed earlier that prolonged oxidative stress can attenuate adaptive AMPK signaling and inhibit autophagy. We uncovered consensus NRF2 binding sites in both the promoter region and the first intron of the human AMPK gene. One putative binding site is located 100 bp upstream and the other 2.2 kb downstream of the ATG translational initiation site. We hypothesized that during oxidative stress, possible NRF2-dependent suppression of autophagy by down-regulating the AMPK signaling pathway may be a strategy to control cell survival.

**Figure 1:**
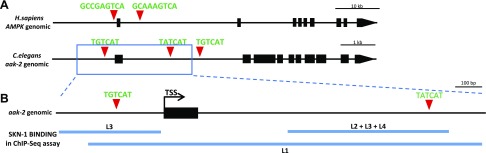
Putative conserved NRF2/SKN-1 binding sites were found in *aak-2/AMPK* genes. *A*) Location and sequences of the conserved putative NRF2/SKN-1 transcription factor binding sites, relative to the transcription start site (+1) in different species. (N denotes arbitrary nucleotides, R indicates adenine or guanine, and S indicates cytosine or guanine.) *B*) The genomic region of AMPK and *aak-2* gene with predicted NRF2 and SKN-1 binding sites. Black boxes correspond to exonic sequences; connecting lines represent introns. Red triangles indicate conserved NRF2/SKN-1 binding sites. *C*) The genomic region of the *aak-2* gene with 2 possible SKN-1 binding sites. The blue lines indicate the genomic regions where Brdlik *et al.* (44) identified binding sites for SKN-1 in their chromatin immunoprecipitation and multiplex sequencing (ChIP-Seq**)** assay in any of the 4 larval stages of *C. elegans*. Black boxes correspond to exons; connecting lines represent introns. Red triangles indicate conserved SKN-1 binding sites. TSS indicates the transcription start site. L1, 2, 3, and 4 are the 4 larval stages of *C. elegans* before reaching adulthood.

The canonical NRF2/ARE binding sites are present in the proximity of *AMPK* genes of other vertebrate and invertebrate species, including *C. elegans* (**Fig. 1*A, B***). Our genome-wide sequence analysis identified 3 canonical SKN-1 (NRF2 ortholog) binding sites (WWTRTCAT, where R = A or G) in the regulatory and coding regions of the *C. elegans aak-2* gene (worm ortholog of *AMPK*), 100 bp upstream and 1073 and 1573 bp downstream of the ATG translational initiation site, respectively. These sites are also highly conserved in the *aak-2* genomic region of closely related *Caenorhabditis* species (Fig. 1*A*). Next, we compared these *in silico* predicted SKN-1 binding sites with the experimentally identified regulatory elements found in the relevant analysis of the model organism Encyclopedia of DNA Elements (modENCODE) project (44). According to a chromatin immunoprecipitation and multiplex sequencing analysis (Brdlik *et al.*)(44), the second binding site of SKN-1 seems most likely to be functional, as SKN-1 protein was found to bind to this region of the *aak-2* gene in every larval stage tested (L1–L4) (Fig. 1*C*). SKN-1 was also found to bind to the promoter region of *aak-2* but not at every larval stage. These results suggest that these ARE sites might be functional and that the second potential binding site is most likely to be an actual ARE. These data, together with the presence of conserved NRF2/SKN-1 binding sites in the *AMPK/aak-2* locus, raise the possibility that putative molecular interactions between NRF2/SKN-1 and *AMPK/aak-2* are evolutionarily conserved from worms to humans.

### NRF2 represses *AMPK* expression

To analyze the regulatory effect of NRF2 on *AMPK* expression during oxidative stress, relative levels of both *AMPK* and *NRF2* mRNA were measured with qPCR under oxidative stress–induced circumstances. We established a protocol to induce oxidative stress in HEK293T cells by using the organic peroxide TBHP. TBHP is a widely used agent that results in oxidation of reduced glutathione, peroxidation of cellular lipids, and altered Ca^2+^ homeostasis, which leads to cell death (45). The oxidizing effect of TBHP was verified by using flow cytometry (Supplemental Fig. S1). TBHP induced ROS production and lipid peroxidation (see the table and histogram red line: 300 μM TBHP on Supplemental Fig. S1) as opposed to untreated cells (table and histogram black line with gray background on Supplemental Fig. S1) as measured by increased DCF and BOD fluorescence, respectively. Silencing of *NRF2* through attenuated both TBHP-induced ROS production and lipid peroxidation (table and histogram green line: 300 μM TBHP + NFκB siRNA on Supplemental Fig. S1), but an oxidative environment was observed.

**Figure 2:**
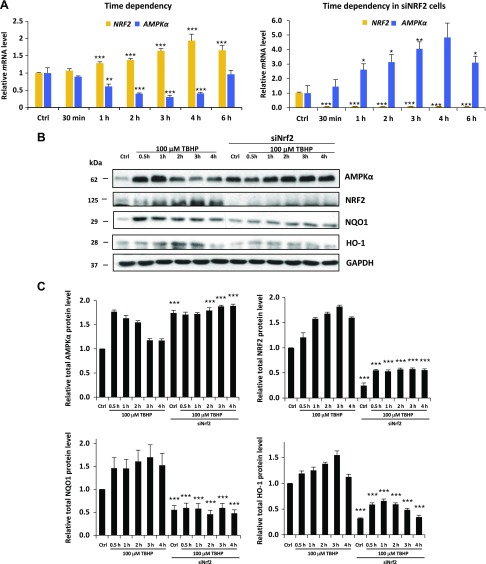
NRF2 down-regulates both mRNA and protein levels of AMPK in the HEK293T cell line after long exposure to oxidative stress. *A*) Time dependency of both *NRF2* and *AMPK* mRNA levels in oxidative stress with/without silencing of *NRF2* by siRNA. HEK293T cells were treated with 100 µM TBHP. The relative level of mRNA was measured by quantitative real-time PCR. *Nrf2* gene expression was depleted by *Nrf2* siRNA. TBHP-treated samples were compared with control (Ctrl) samples. *B*) Time dependency of total AMPK protein levels (AMPK-T), NRF2, NQO1, and HO-1 during oxidative stress. Cells were treated with 100 µM TBHP, while the AMPK level was detected by immunoblotting. *Nrf2* gene expression was depleted by *Nrf2* siRNA. GAPDH was used as a loading control. *C*) Quantification and statistical analysis of Western blot assays. Densitometry data represent the intensity of AMPK-T, NRF2, NQO1, and HO-1 normalized for GAPDH. Samples were compared with their partner with siNRF2 background. Error bars represent ± sem. *P < 0.05, **P < 0.01, ***P < 0.005 (independent 2-sample Student’s *t* tests).

Generating oxidative stress by adding various concentrations of TBHP clarified that 1.5 h of treatment with 100 µM TBHP was able to increase the level of *NRF2* mRNA, whereas addition of 300 µM TBHP resulted in a slight decrease in the *NRF2* mRNA level in control cells (**Fig. 2** and Supplemental Fig. S2). Due to the fact that NRF2 is mainly controlled at the posttranscriptional level, we also analyzed NRF2 activity on the protein level. In addition to immunoblotting NRF2, two well-known NRF2 targets, NQO1 (46) and HO-1 (47), were used to follow the time-dependent activation of NRF2 on a protein level. Therefore, we chose 100 µM TBHP treatment to focus on the time dependency of oxidative stress in HEK293T.

We found that the effect of TBHP treatment on *AMPK* and *NRF2* mRNA levels occurs in a time-dependent manner (Fig. 2*A*). The longer the cells were treated with TBHP, the more *NRF2* and less *AMPK* mRNA were present in cells. In cells growing for 1, 2, 3, or 4 h in 100 µM final concentration, TBHP medium was significantly less *AMPK* (at 4 h, only 41% mRNA relative to control cells) and more *NRF2* mRNA (at 4 h 194% mRNA relative to control cells). These qPCR analysis results suggest that oxidative stress lowers the transcription of *AMPK* with a certain time delay while quickly enhancing the transcription of *NRF2*. Interestingly, the *AMPK* mRNA level did not show any decrease when TBHP treatment was preceded by using siRNA to silence NRF2. The fact that lowering of *AMPK* mRNA levels requires NRF2 suggests a regulatory interaction between the *AMPK* gene and NRF2 protein.

To verify the relationship between *AMPK* and oxidative stress, the expression level of total AMPK proteins was analyzed by using Western blot in HEK293T cells. Time dependency was clearly shown in AMPK levels because they were elevated (from 4 to 9 times) in each sample treated with TBHP (1–4 h) relative to the control samples (Fig. 2*B, C* and Supplemental Table S1). Both AMPK phosphorylation and its total protein level showed a transient peak during TBHP treatment, and this activation profile is correlated to NRF2, NQO1, and HO-1 level supposing that NRF2 might have some regulatory role in *AMPK*. The densitometry data of AMPK protein level normalized for GAPDH directly show us that the AMPK protein level was also decreased after 3 h of TBHP treatment. It is well known that the half-life of AMPK is relatively long; therefore, the AMPK protein level could remain high much longer compared with the AMPK mRNA level during permanent oxidative stress. To further analyze the effect of NRF2 on *AMPK* during oxidative stress, Western blot experiments were also performed when NRF2 was silenced by using siRNA. NRF2 silencing was detected by depletion of NRF2, NQO1, and HO-1 protein levels. Relative to control cells, AMPK levels were significantly increased in cells treated with siNRF2. In these cells, AMPK protein levels were not suppressed; however, a 7 times higher basic AMPK level was present in control and treated cells relative to the unsilenced samples. These data suppose that oxidative stress–induced NRF2 is able to suppress human AMPK levels in both a time- and dose-dependent manner.

### NRF2 directly binds to the ARE binding site at AMPK

**Figure 3:**
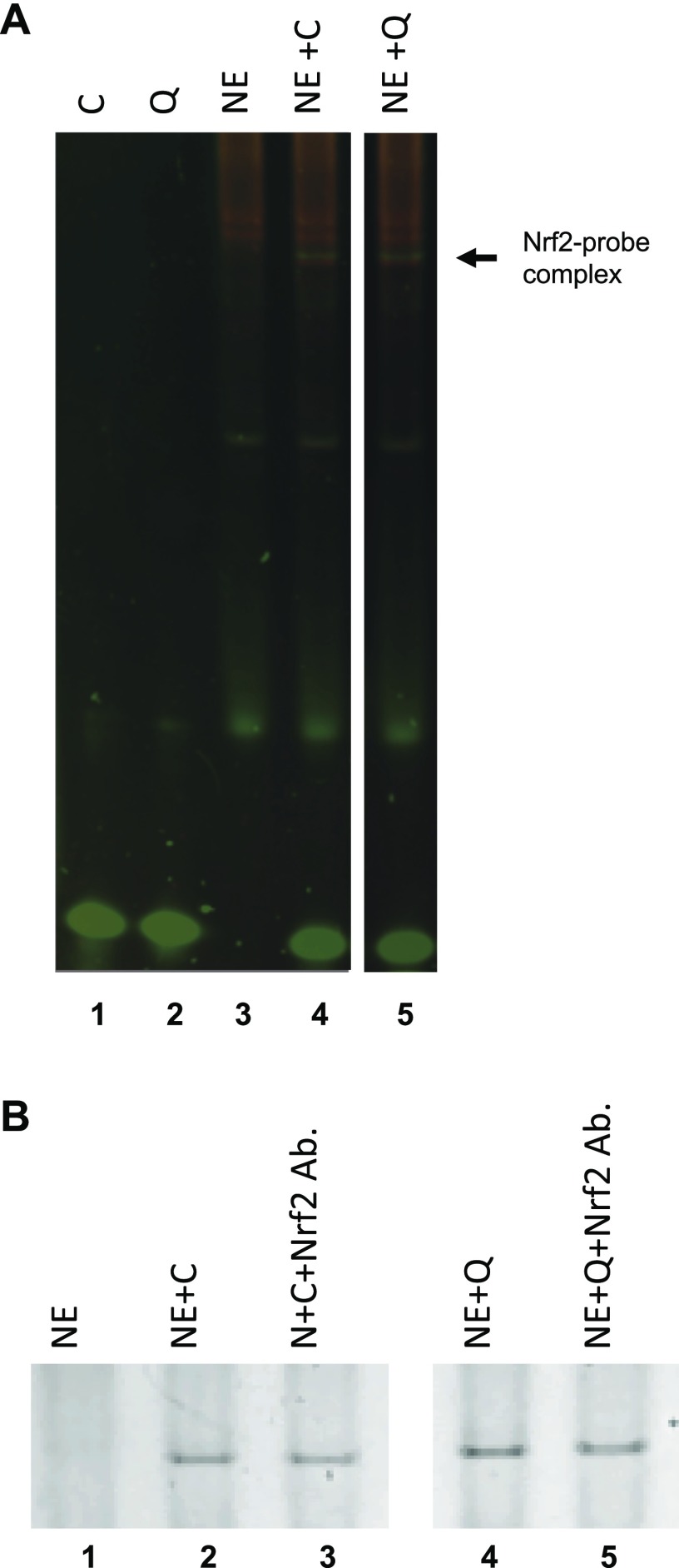
NRF2 binds to AMPK query and consensus oligonucleotide sequences. Nuclear extracts (NE) of Caco-2 treated with TBHP were incubated with query (Q) (lane 4) or consensus (C) (lane 5) oligonucleotide probes and compared with nuclear extract only control (lane 1). Image of the EMSA gel stained with SYBR Green EMSA stain identified nucleic acids (green), and subsequent SYPRO Ruby EMSA stain identified protein (red) components. NRF2 protein–probe complexes (yellow) formed only in lanes containing nuclear extracts + probe. *B*) Caco-2 nuclear extracts were incubated with 2 μg of anti-NRF2 antibody (Ab.) before the addition of oligonucleotide probe (lanes 3 and 5) and analyzed for complex retardation.

To assess whether NRF2 is able to bind to its potential ARE sequence in the promoter region of *AMPK* (query sequence), an EMSA study was performed with nuclear extracts isolated from Caco-2 cells treated with TBHP for 4 h. We used the well-known, consensus ARE sequence as a positive control. Assembly of the NRF2 protein–probe complex was visualized by using a 2-color fluorescence staining technique to observe both the nucleic acid and the protein components of the assay (48). As shown in **Fig. 3*A***, the gel shift assay identified protein–probe complex formation in the presence of either AMPK query (lane 5) or consensus (lane 4) oligonucleotides, compared with no complex formation in control lanes containing oligonucleotide probe (lanes 1–2) or nuclear extract only (lane 3). Supershift assays using a specific anti-NRF2 antibody showed a 11 and 10.5% decrease in the protein–probe complex formation for both the AMPK query and the consensus oligonucleotide probes, respectively (Fig. 3*B*), indicating the presence of NRF2 in the protein–probe complex. Although no retarded NRF2 complex was identified, it has been shown that binding of the antibody to NRF2 protein can reduce its oligonucleotide probe-binding capacity (49). These results indicate NRF2 binds to both AMPK query and consensus oligonucleotide sequences.

### NRF2 negatively regulates prolonged autophagy *via* AMPK

**Figure 4:**
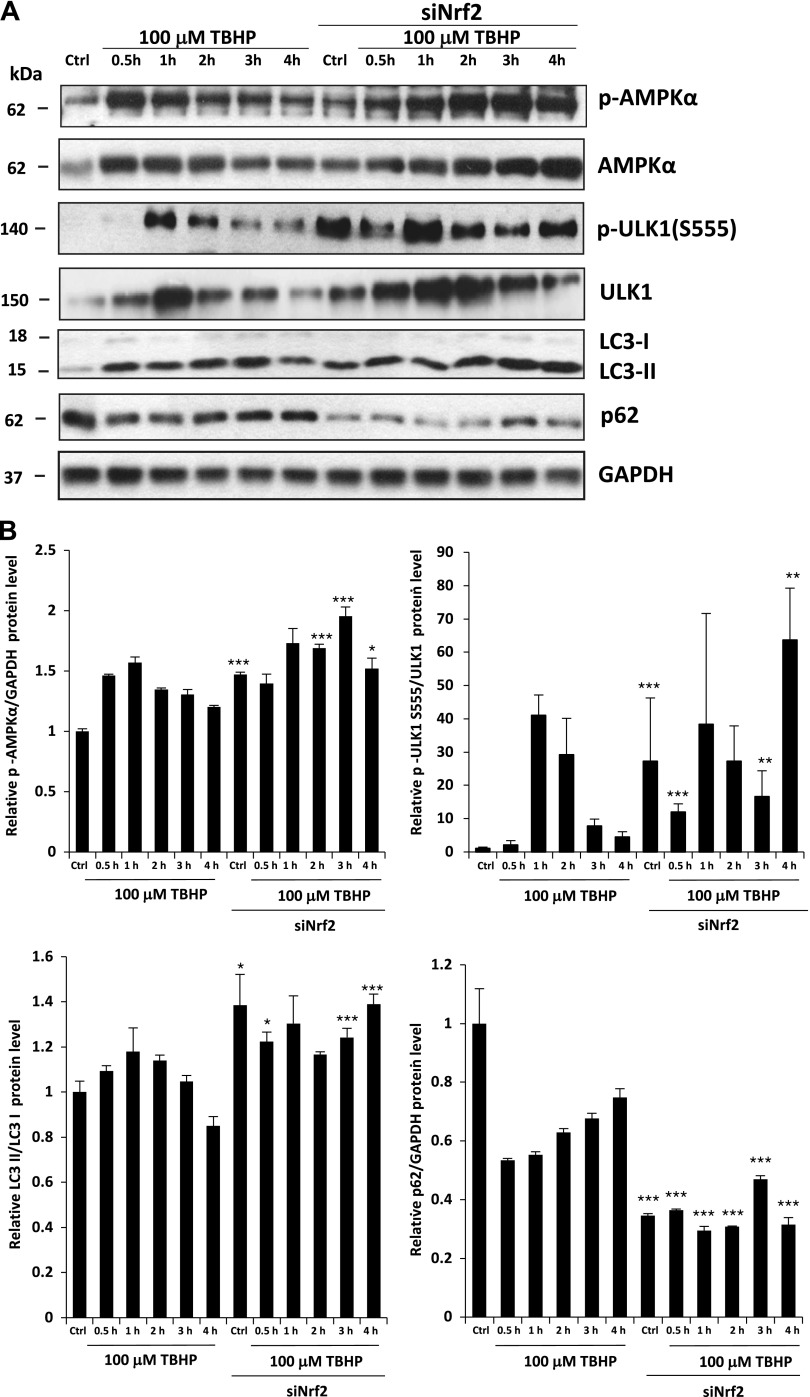
NRF2 down-regulates autophagy proteins after long exposure to oxidative stress in the HEK293T cell line. *A*) Time dependency of autophagy down-regulation by NRF2 in oxidative stress. Western blot results of HEK293T cells in 100 µM TBHP-containing media. The Western blot shows the time course levels of autophagy-related proteins. Cells were cultured with TBHP for 0.5, 1, 2, 3, and 4 h, while *NRF2* gene expression was depleted by *NRF2* siRNA. GAPDH was used as a loading control. *B*) Quantification and statistical analysis of the Western blot assays. Densitometry data represent the intensity of p62 normalized for GAPDH, LC3-II normalized for LC3-I, ULK555-P normalized for total level of ULK, and AMPK-P normalized for GAPDH. Samples were compared with their partner with siNRF2 background. Error bars represent ± sem. *P < 0.05, **P < 0.01, ***P < 0.005 (independent 2-sample Student’s *t* tests).

To address the impact of oxidative stress and NRF2 on autophagy, we also analyzed the time dependency of certain key autophagy markers in TBHP treatment by immunoblotting in HEK293T cells (**Fig. 4**). To detect whether AMPK is activated upon oxidative stress, the level of active, phosphorylated AMPK was measured. Because not only does the level of p-AMPKAalpha change upon oxidative stress but AMPKAalpha is also up-regulated, on Fig. 4*B* we show the time profile of p-AMPKAalpha/GAPDH during oxidative stress. We also analyzed the phosphorylation status of its direct target, ULK1. Phosphorylated Ser555 on ULK1 assumes an activated autophagy process induced by phospho-AMPK. Interestingly, we found a transient activation for AMPK upon prolonged oxidative stress. Both AMPK and ULK555 were fully phosphorylated after 1 h of TBHP treatment, suggesting an essential role of AMPK in autophagy induction. However, AMPK activity later quickly dropped (as indicated by its dephosphorylation after 3 h of TBHP treatment), suggesting a delayed negative feedback on AMPK activity during oxidative stress. Some key autophagy markers (*e.g.*, LC3, p62) also indicated that autophagy exhibits an activation peak during oxidative stress, but long treatment with an oxidative agent suppresses autophagy. To confirm that this time profile of autophagy activation occurs in an NRF2-dependent manner, the experiment was repeated in NRF2-silenced HEK293T cells. Remarkably, both AMPK-P and ULK555P were increased in *NRF2*-silenced control cells. Furthermore, levels of each autophagy markers we used (AMPK-P, ULK555P, and LC3-II) remained high, whereas the autophagy substrate p62 was robustly reduced after 4 h of TBHP treatment in cells pretreated with *NRF2* siRNA. These results confirm that NRF2 negatively regulates autophagy *via* AMPK.

**Figure 5:**
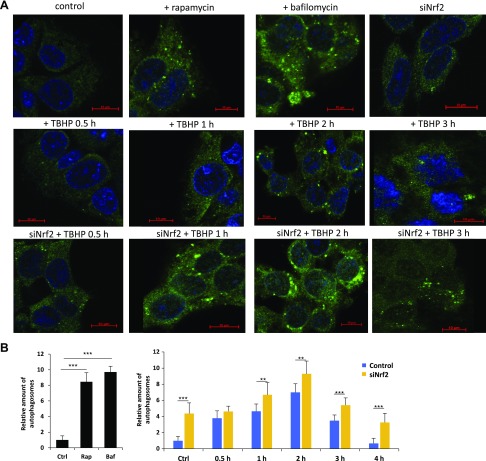
*NRF2* silencing positively regulates autophagy. The connection of NRF2-dependent oxidative stress and autophagy induction was checked by immunofluorescence microscopy. LC3 was stained by green fluorescence dye (Alexa Fluor 488); therefore, the green dots in the cells represent functional autophagosomes. *A*) Time dependency of TBHP-induced (100 μM) autophagy regulation by NRF2. Rapamycin (Rap, 100 nM, 2 h) and bafilomycin (Baf, 10 μM, 2 h) were used as positive controls. *B*) Quantification and statistical analysis of immunofluorescence microscopy data. Error bars represent ± sem. **P < 0.01, ***P < 0.005 (independent 2-sample Student’s *t* tests).

Densitometry data of autophagy markers (*i.e.*, LC3-II/LC3I, p62, ULK555) show that autophagy has a transient activity change during oxidative stress (Fig. 4*B*). To verify the delayed negative effect of NRF2 on autophagy with respect to permanent oxidative stress, autophagy was analyzed by using immunofluorescence microscopy (LC3 ICC staining) with and without NRF2 silencing (**Fig. 5**). To generate oxidative stress, TBHP was added to HEK293T cells for several hours prior to with/without *NRF2* siRNA treatment. Cells were fixed and immunolabeled for endogenous LC3-I/II (green). Thus, LC3-I shows diffused cytoplasmic staining, and LC3-II can be observed in discrete foci. As positive controls, bafilomycin A1 and rapamycin were used. Although bafilomycin A1 is a well-known inhibitor of the late phase of autophagy resulting in the latent accumulation of autophagosomes, rapamycin hyperactivates autophagy *via* mTOR inhibition (50–53). We detected a 5-fold increase in the relative amount of autophagosomes in control cells treated with TBHP; however, after 4 h of treatment, the amount of autophagosomes was similar to the basal level. In contrast, the relative amount of autophagosomes remained significantly high both in the absence of NRF2 under control conditions and during excessive levels of TBHP. These results fit to the Western blot analysis and confirm at the cellular level that transient autophagy is down-regulated by NRF2 upon prolonged oxidative stress (Supplemental Fig. S3).

### *In vivo* evidence for the relationship of *aak-2* and SKN-1

**Figure 6:**
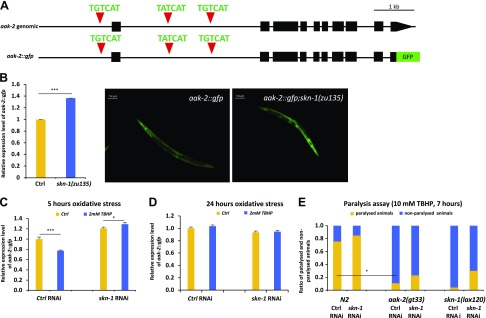
SKN-1/NRF2 down-regulates *aak-2/AMPK* expression upon oxidative stress in *C. elegans. A*) The genomic region of *aak-2* with predicted SKN-1 binding sites, and the structure of the translational *aak-2::gfp* reporter gene, is shown. Black boxes correspond to exonic sequences, connecting lines represent introns. Red triangles indicate conserved SKN-1 binding sites. *B*) Expression of *aak-2::gfp* transgene was increased in *skn-1(-)* mutant background. *C*) In acute oxidative conditions (2 mM TBHP, 5 h), *aak-2::gfp* expression was decreased in animals with wild-type background. The absence of *skn-1* gene products eliminated this change of gene expression. *D*) In chronic oxidative conditions (2 mM TBHP, 24 h), *aak-2::gfp* gene expression was not inhibited. *E*) Paralysis assay of worms held on 10 mM final concentration TBHP plates. Data are expressed as the ratio of paralyzed/nonparalyzed worms. The number of animals examined: *n* = 15–100. Error bars represent ± sem. All experiments were independently reproduced 3 times. *P < 0.05, ***P < 0.005 (independent 2-sample Student’s *t* tests).

As a model organism, *C. elegans* provides several characteristics that can confirm our *in vitro/in silico* and cellular models. The conservation of disease/signaling pathways between *C. elegans* and higher organisms makes the worm an effective *in vivo* model. We used an *aak-2::gfp* reporter strain (39) and tested how its expression is changed in response to oxidative stress. The translational fusion reporter construct contains the whole genomic region of *aak-2* gene and the 2 kb upstream promoter region (**Fig. 6*A***). Broad GFP expression was observed in the pharynx, gut, and nervous and reproduction systems of the young adult animals. The reporter was up-regulated in *skn-1*(−) null mutants (*P* < 0.005) (Fig. 6*B*), as well as in *skn-1* RNAi-treated animals (*P* < 0.0001) (Supplemental Fig. S4). The most apparent/striking differences regarding the intensity of fluorescence were observed in the head and pharynx of the animals. In acute oxidative stress (5 h), the *aak-2::gfp* expression was decreased (*P* < 0.0001), but this reduction in fluorescence disappeared when the *skn-1* gene was silenced (Fig. 6*C*). The inhibition of *aak-2::gfp* expression by oxidative stress was dose dependent: we observed the maximal inhibition at 2 mM TBHP concentration (Supplemental Fig. S5 and Supplemental Table S2). However, this decrease of *aak-2::gfp* expression was suppressed when worms were maintained on TBHP-containing plates for 24 h (Fig. 6*D*). Altogether, these results provide *in vivo* evidence for the time- and dose-dependent relationship of *aak-2* expression, oxidative stress, and SKN-1.

To test the role and the position of *aak-2* and *skn-1* in oxidative stress response, we measured the oxidative resistance of *skn-1* RNAi-treated and/or *aak-2 and skn-1* mutant worms. In response to higher level of oxidative stress (10 mM TBHP), *C. elegans* displays loss of motility and progressive paralysis. Without SKN-1, this paralysis is more definite after 7 h (76% of paralyzed animals in wild type *vs.* 85% in SKN-1 depleted animals) (Fig. 6*E* and Supplemental Table S3). Eleven percent of the *aak-2(gt33)* deletion mutant animals were paralyzed after 7 h, whereas 76% of the N2 animals lost their motility. Without SKN-1, this difference was smaller (N2 *vs.* *aak-2(gt33)*: 85 *vs.* 23%). Most of the *skn-1(lax120)* gain-of-function mutant animals remained mobile even after 7 h, but this resistance to oxidative stress was suppressed in *skn-1* silenced animals. *skn-1* gain-of-function mutants displayed TBHP resistance reminiscent of *aak-2* loss-of-function mutants. Thus, SKN-1 is likely to promote TBHP resistance by negatively regulating *aak-2* activity.

In *C. elegans*, tagging LGG-1 (worm ortholog of Atg8/LC3) with a fluorophore has become a widely accepted method to visualize autophagosomes (54, 55). The quantitative assessment of the fluorescence signal of cells with numerous LC3-positive puncta can be assessed accurately at a single-cell level (56). GFP::LGG-1 is expressed throughout development in multiple tissues and displays a diffuse expression pattern, whereas under conditions that induce autophagy, GFP::LGG-1 labels positive punctate structures, and its overall expression increases in multiple tissues, including intestinal cells. The amount of GFP::LGG-1–positive vesicles correlates with the formation of autophagosomes, making GFP::LGG-1 an acceptable marker for detecting autophagosomes. Thus, we could test whether AAK-2 or SKN-1 is required for autophagy by monitoring changes in the number of GFP-positive structures or the ratio of GFP-positive area (autophagosomes) in the cells of transgenic animals (57, 58).

**Figure 7:**
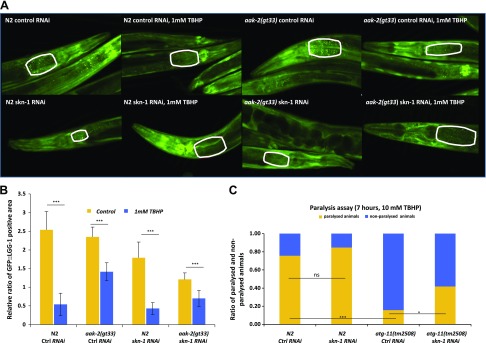
Oxidative stress decreases the level of autophagy. *A*) Fluorescent images showing GFP-tagged LGG-1 accumulation in control animals *vs.* in animals treated with 1 mM TBHP for 3 h. In one half of the animals, the *skn-1* gene expression was depleted by *skn-1* RNAi. GFP::LGG-1 accumulates in punctuate areas that are supposed to indicate autophagosomal structures. *B*) Quantification and statistical analysis of fluorescent images of GFP::LGG-1 foci in individual cells. GFP::LGG-1–positive dots that are believed to correspond to autophagosomal structures were quantified. *C*) Paralysis assay of worms held on 10 mM final concentration TBHP plates. Data are expressed as ratio of paralyzed/nonparalyzed worms. *skn-1* gene expression was depleted by *skn-1* RNAi. The number of animals examined: *n* = 40–70. Error bars represent ± sem. All experiments were independently reproduced 3 times. Detailed statistics for each assay can be found in the Supplemental Data. Ns, not significant. *P < 0.05, ***P < 0.005 (independent 2-sample *t* tests).

To further analyze the possible relationship between oxidative stress, SKN-1, AAK-2, and autophagy, we also measured relative autophagy activity in *aak-2* mutant worms. We incubated the young adult animals on plates containing 1 mM TBHP for 3 h with or without *skn-1* RNAi treatment, using an integrated GFP-tagged LGG-1 reporter strain our group generated earlier (59). There was no significant autophagy activity difference in the control RNAi N2 and *aak-2* mutant animals without TBHP treatment. Upon oxidative stress, the area of GFP::LGG-1–positive loci decreased drastically by 80% (**Fig. 7*A, B*** and Supplemental Table S4) in wild-type animals (*P* < 0.0001). In *aak-2*(*gt33)* mutant worms, however, this reduction was not as definite [only 40% (*P* < 0.0001)]. This finding means that in animals lacking functional AAK-2 protein, the effect of oxidative stress on autophagy is suppressed. If *skn-1* gene expression is depleted, autophagy decreased by 33% (*P* < 0.0001) in the cells of the wild-type animals. However, in TBHP-treated *aak-2* mutants, the ratio of GFP-positive area of autophagosomes did not decrease as much as in wild-type animals (76 *vs.* 58% reduction in TBHP treated *N2* *vs.* *aak-2(gt33)* worms, respectively) (Fig. 7*B* and Supplemental Table S4). According to this result, in the presence of the *aak-2* mutation, autophagy is more active during oxidative stress. Oxidative stress lowers the level of autophagy both in wild-type and in *aak-2* mutant animals, but this effect is smaller if the *skn-1* gene is silenced.

We also examined the resistance/tolerance of autophagy deficiency upon oxidative stress by using *atg-11* mutant nematodes [*tm2508* allele containing a 548 bp deletion (*atg-11* has a role in the initiation of autophagosome formation)]. After 7 h of 10 mM TBHP treatment, 76% of the wild-type animals became paralyzed (Fig. 7*C* and Supplemental Table S5), whereas only 16% of autophagy mutant *atg-11* animals exhibited a paralyzed phenotype. Thus, the stress resistance/tolerance of the autophagy-deficient mutant animals is better than the wild-type’s during oxidative stress, indicating that autophagy could not be beneficial upon long-term oxidative stress. The presence or absence of *skn-1* gene products changed this ratio only in the *atg-11* mutant animals. With these *in vivo* experiments, we confirmed that the effect of SKN-1 on autophagy occurs through the inhibition of *aak-2* expression, showing that this down-regulatory mechanism is well conserved between worms and humans.

## DISCUSSION

NRF2, as a key antioxidant response regulator, and autophagy, as an important cellular mechanism to eliminate ROS and reactive nitrogen species, act together to promote cell survival upon oxidative stress (3, 32, 60). ROS and oxidative stress/damage alter autophagic flux rates, and excessive autophagy can promote cell death through the degradation of important components within the cell (32, 61). However, if oxidative stress is prolonged or already contained, we showed that it is also NRF2 that acts as a master regulator to turn off autophagy to avoid the excessive destruction of cellular components, and possibly to save the cell from autophagic cell death (32). Our results show that NRF2 binds to both AMPK query and consensus oligonucleotide sequences, suggesting that AMPK can be directly regulated by NRF2. We predicted and confirmed an evolutionary conserved mechanism by which NRF2 regulates the expression of the autophagy inducer AMPK, and, by this action, decreases the activation of autophagy to physiologic levels. We also presented that the NRF2-mediated autophagy down-regulation can only be observed upon prolonged stress, highlighting why this exciting mechanism was not yet discovered. Because both the function of NRF2 and the regulation of autophagy are highly complex and context dependent (6, 41, 62), we cannot exclude other important factors in relation to NRF2, AMPK, and autophagy. Here, we highlighted a seamless but effective molecular mechanism for how the antioxidant master regulator NRF2 turns off autophagy when autophagy has performed its function.

Exploring the regulatory network of NRF2, we proposed a model of a direct transcriptional regulation between NRF2 and the autophagy regulator AMPK. Many experimental data have shown that AMPK can stimulate NRF2 signaling during oxidative agents (*e.g.*, hydrogen peroxide) (30, 63). The detailed mechanism of this regulatory connection, however, is yet unknown. NRF2 promotes autophagy through p62. Because *NRF2* knockout reduces cell viability, we can assume that NRF2 has a crucial role in promoting autophagy-dependent survival during oxidative stress. In the present study, we confirmed that NRF2 was able to bind the ARE binding site at *AMPK* (Fig. 3), suggesting that NRF2 directly regulates the expression of AMPK. We proved this binding by EMSA; however, this connection needs further confirmation to show more direct proof for NRF2 binding to ARE elements (*e.g.*, by using chromatin immunoprecipitation analysis). We also described that NRF2 is able to down-regulate *AMPK*, and this negative effect was manifested at the transcriptional level during prolonged cellular stress in both the human cell line and *C. elegans* (Figs. 2*A, B* and 6*B, C*). By following in time the level of main components with respect to TBHP-induced oxidative stress, it turned out that the timing has an important role in this network (Figs. 2*A, B* and 6*C, D*). Namely, AMPK quickly responds to oxidative exposure. On the one hand, AMPK becomes active *via* phosphorylation; on the other hand, the AMPKAalpha protein amount is also increased. The active AMPK can activate NRF2. This regulatory loop implies that at the very beginning of oxidative stress, AMPKAalpha protein abundance is independent of NRF2 effects. In addition, this fast response leads to autophagy induction. The autophagy markers (*i.e.*, LC3-II/LC3-I and p62) illustrate (Fig. 4*B*) that autophagy has a transient activity change during oxidative stress. LC3-II level was quickly induced, whereas the p62 level was decreased when TBHP was added to HEK293T cells. The LC3-II/LC3-I level started to decrease, while the p62 level increased back after 3 h of oxidative stress. The key autophagy activator ULK1 has also shown a transient activatory phosphorylation on its Ser555, further supposing a transient activation of autophagy. This result seems to fit to microscopy data when the LC3-II–positive foci were detected upon oxidative stress (Fig. 5). Both data confirm that autophagy has a delayed down-regulation during permanent TBHP treatment. However, after a certain time delay, NRF2 is able to down-regulate *AMPK*. Down-regulation of *AMPK* results in the inactivation of its targets (including NRF2). The silenced *NRF2* gene results in the continuous up-regulation of AMPK during extended oxidative stress because the delayed negative feedback has been diminished from the control network.

The *C. elegans* experiments performed here have further uncovered this important regulatory mechanism of autophagy and confirmed the *in vitro* results. In the present study, we found that in response to oxidative stress, SKN-1 down-regulates *aak-2* in *C. elegans* as well. Relative to control animals, *aak-2::gfp* expression levels were significantly increased in animals treated with *skn-1* RNAi. SKN-1 was required to keep *aak-2* expression at low levels upon acute oxidative stress, whereas this effect was not seen under chronic oxidative stress circumstances. We noted that SKN-1 down-regulates *aak-2* (Fig. 6*C, D*) not only as part of the antioxidant response but under normal circumstances as well. Similarly, >60 genes were previously described as being down-regulated under basal conditions by SKN-1 (64, 65). In the paralysis assay, the *aak-2* mutant animals tolerated oxidative stress better than the wild-type animals. However, this increased stress tolerance seemed SKN-1 dependent (Fig. 6*E*). The decreased stress tolerance of the *skn-1* RNAi-treated *skn-1* gain-of-function mutant animals (relative to the control *skn-1* mutant animals) proves that the gene silencing with RNAi worked, and it was effective. Animals carrying the *gain-of-function* mutation display high stress resistance among. The reason for the elevated but not significant increase (4 *vs.* 30% paralyzed animals) of stress tolerance of the *skn-1* gain-of-function mutant animals (compared with *skn-1* RNAi animals) may be a function of the not completely *skn-1* gene-silenced neuronal cells. In these cells, some SKN-1 protein is constitutively active and can influence motility despite the RNAi.

**Figure 8:**
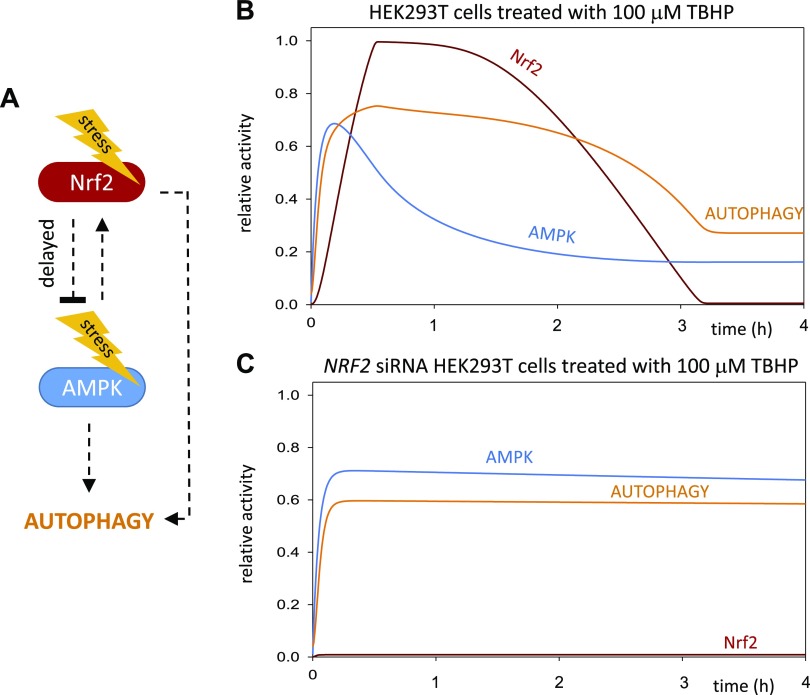
Proposed computational model of NRF2-dependent AMPK down-regulation during oxidative stress. *A*) The core network of AMPK and NRF2 regulation with respect to oxidative stress. The dotted line denotes how the components can influence each other. *B*) Numerical simulations of relative protein levels and activities of the HEK293T cell line with a mathematical model during oxidative stress. Both AMPK and NRF2 have a transient activation profile resulting in the down-regulation of autophagy after 4 h of oxidative stress. *C*) Numerical simulations of relative protein levels and activities of the HEK293T + siNRF2 cell line with a mathematical model during oxidative stress. The active AMPK is able to maintain the autophagic process high in the absence of NRF2.

We believe that this delayed negative feedback between AMPK and its targets plays a significant role in the fine-tuning of autophagy induction. To further explore the role of NRF2 in the delayed down-regulation of AMPK during oxidative stress in human cell line, we propose a theoretical mathematical model to illustrate and interpret the important connections and feedback of the NRF2/AMPK regulatory network (**Fig. 8*A***). According to our experimental data, AMPK has a quick transient activation followed by autophagy induction with respect to oxidative stress (Fig. 8*B*). After 4 h of treatment with an oxidative stressor, NRF2-dependent down-regulation of *AMPK* occurred, controlled by a delayed negative feedback between AMPK and NRF2. This connection immediately induces the inactivation of autophagy during prolonged oxidative stress. A quick activation of autophagy is essential with respect to oxidative stress because the bulk of damaged components have to be reduced during oxidative stress. Obviously, the extended autophagic event is not beneficial for the cellular system; therefore, the cell tries to reduce autophagy to avoid a harmful self-digestion. We propose that this delayed negative feedback between AMPK and NRF2 has a main role in guaranteeing that autophagy does not become hyperactivated even at high levels or prolongation of cellular stress. In the absence of NRF2, the delayed negative feedback becomes disrupted; therefore, the AMPK levels remain high even after 4 h of treatment with an oxidative stressor (Fig. 8*C*). The high level of AMPK is able to keep the autophagy processes active. Our results suggest that for the proper cellular stress-dependent activation of autophagy, both AMPK and NRF2 are required. At an early stage of the autophagy process, AMPK is required for autophagy activation, while in the later phase of the stress event, NRF2 is essential to down-regulate the process *via* inhibiting its own activator. In this way, with certain delay, NRF2 start down-regulating AMPK, and therefore, the level of active autophagy is also dropping. However, in our model, we did not rule out that NRF2 has a positive effect on autophagy induction in later phases of oxidative stress. We believe that NRF2 alone is not sufficient to maintain autophagic response when AMPK is diminished.

In healthy cells, stress induces autophagy; however, above a certain level, autophagy could result in cell death or may lead to the onset of harmful processes (1, 32, 66). Thus, we propose that SKN-1 is supposed to prevent these undesired cellular events. Elevated levels of autophagy were detected (in an *aak-2*–dependent manner) in animals exposed to oxidative stress when SKN-1 protein was depleted (Fig. 7*A, B*). This is not the first known regulatory mechanism through which SKN-1 controls autophagy. SKN-1 regulates autophagy through binding to the ARE element in the promoter of *p62* gene as well, and p62 in turn delivers polyubiquitinated cargoes to autophagy *via* the LIR/Atg8 family–interacting domain (17, 25).

In summary, we established a new role for NRF2 in regulating the dynamics of autophagy. Upon prolonged oxidative stress, NRF2 decreases the activation of autophagy through the down-regulation of AMPK to prevent the harmful hyperactivation of autophagy, and with that, promote cellular survival. Oxidative stress has been recognized as a major contributing factor in aging and in various forms of pathophysiology, including neurodegeneration, diabetes, liver diseases, and cancer (67–75). In the last decade, the role of autophagy in oxidative stress–related diseases has been reported in detail, showing that normally functioning autophagy can promote cellular survival, whereas malfunction in autophagy regulation can lead to cell death and disease phenotypes. However, the method by which activated autophagy is turned off when oxidative stress is prolonged or already contained has not been elaborated, despite several phenotypic reports on the existence of such a crosstalk. In the present study, combining regulatory network analysis with experimental validation in a model organism, *C. elegans*, and in a human cell line, we reported the critical role of NRF2 in inhibiting autophagy hyperactivation upon prolonged oxidative stress. By unraveling how NRF2/SKN-1 could regulate autophagy upon prolonged oxidative stress, we highlighted an evolutionary conserved mechanism through which cellular mechanisms interact to promote survival.

## Supplementary Material

This article includes supplemental data. Please visit *http://www.fasebj.org* to obtain this information.

## Abbreviations

*aak-2*: *Caenorhabditis elegans* ortholog of AMP-activated protein kinase
ARE: antioxidant response element
*atg-11*: autophagy-related protein 11
BOD: Bodipy-[^11^C]
DCF: dichlorofluorescein-diacetate
GAPDH: glyceraldehyde-3-phosphate dehydrogenase
GFP: green fluorescent protein
HO-1: heme oxygenase 1
KEAP1: Kelch-like ECH-associated protein 1
LC3-II: microtubule-associated protein 1A/1B-light chain 3
LGG-1: *Caenorhabditis elegans* ortholog of mammalian LC3 protein
mTOR: mammalian target of rapamycin
NGM: nematode growth medium
NQO1: NAD(P)H:quinone oxidoreductase 1
NRF2: NF-E2–related factor 2
qPCR: quantitative PCR
RNAi: RNA interference
ROS: reactive oxygen species
siRNA: small interfering RNA
SKN-1: protein skinhead-1
TBHP: *tert*-butyl hydroperoxide
ULK1: Unc-51–like autophagy activating kinase 1
