# Supplementary material for: Suppression of AMPK/aak-2 by NRF2/SKN-1 down-regulates autophagy during prolonged oxidative stress
Source: FASEB J. 2018 Oct 2;33(2):2372–87. doi: 10.1096/fj.201800565RR (PMC6338645; doi:10.1096/fj.201800565RR)
Supplement: Supplementary file 1 [file fj.201800565RR.sf1.pdf]

Suppl. Figure 1

A

Sample	% of cells in DCF+
Control	5.1
Control +siRNA	0.87
100 µM TBHP	5.8
100 µM TBHP +siRNA	5.0
300 µM TBHP	19
300 µM TBHP +siRNA	1.5

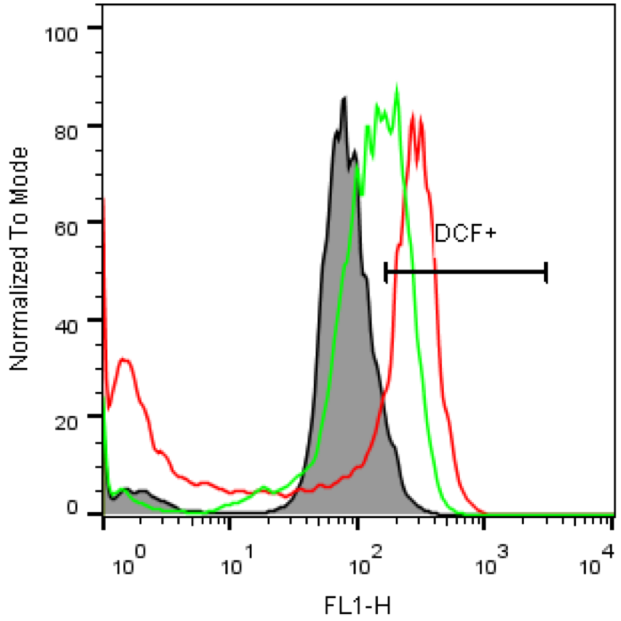

B

Sample	% of cells in BOD+
Control	6.3
Control +siRNA	6.2
100 µM TBHP	25.0
100 µM TBHP +siRNA	17.6
300 µM TBHP	19.9
300 µM TBHP +siRNA	17.4

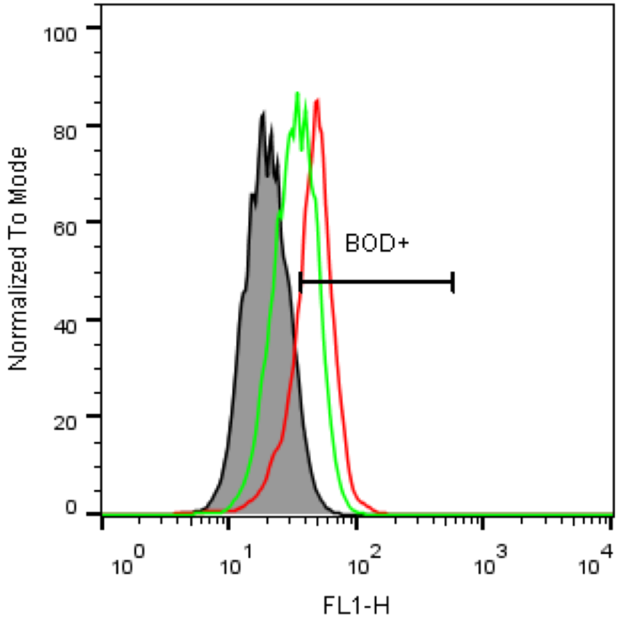

Suppl. Figure 1. Oxidative stress induced by TBHP and the effect of NRF2 siRNA silencing. HEK cells were seeded on 6 well plates and left untreated (Control) or treated as indicated with TBHP (100 or 300 µM) for 2 hours. *NRF2* gene expression was depleted by *NRF2* siRNA. 24 hours before treatment as indicated. After treatment cells were labelled with (a.) 10 µM dichlorofluorescein-diacetate (DCF) or (b.) 2 µM Bodipy-C11 (BOD) and analyzed by flow cytometry. One representative experiment is shown. Black line: untreated cell; red line: 300 µM TBHP; green line: 300 µM combined with NRF2 silencing.

Suppl. Figure 2

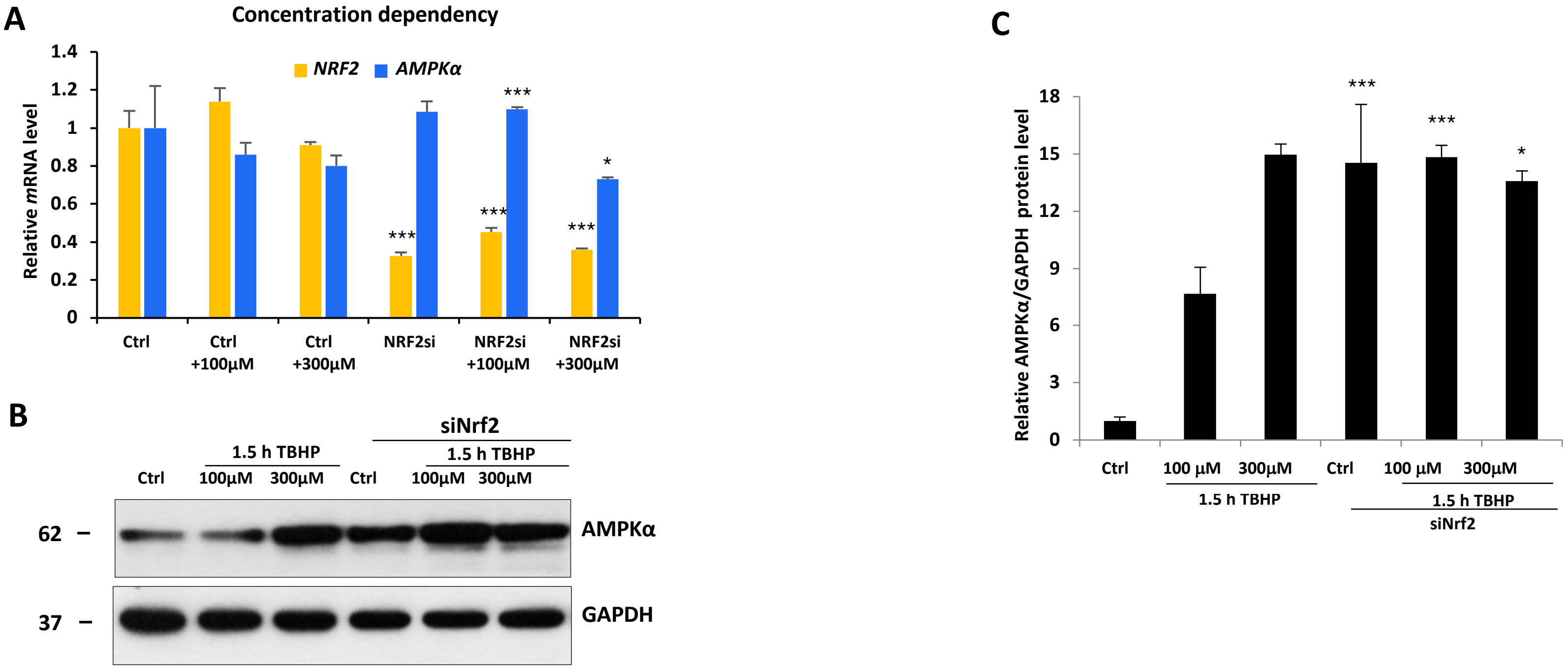

Suppl. Figure 2. NRF2 down-regulates the mRNA and protein level of AMPK in HEK293T cell line. (A) Concentration dependency of both *NRF2* and *AMPKα* mRNA levels in oxidative stress. HEK293T cells treated with 100 or 300 μM TBHP for 1.5 hours. *NRF2* gene expression was depleted by *NRF2* siRNA. Relative level of mRNA was measured by real-time PCR. Samples were compared to their partner with siNRF2 background. **(B)** Concentration dependency of total AMPKα protein level during oxidative stress. Cells were treated with 100 or 300 μM TBHP and AMPKα level was detected by immunoblotting. Nrf2 gene expression was depleted by Nrf2 siRNA. GAPDH was used as a loading control. **(C)** Quantification and statistical analysis of the Western blot assays. Samples were compared to their partner with siNRF2 background. In panels A and C, ***: p<0.005, **: p<0.01, *: p<0.05 (Independent two-sample t-tests). Error bars represent ±SEM.

Suppl. Figure 3

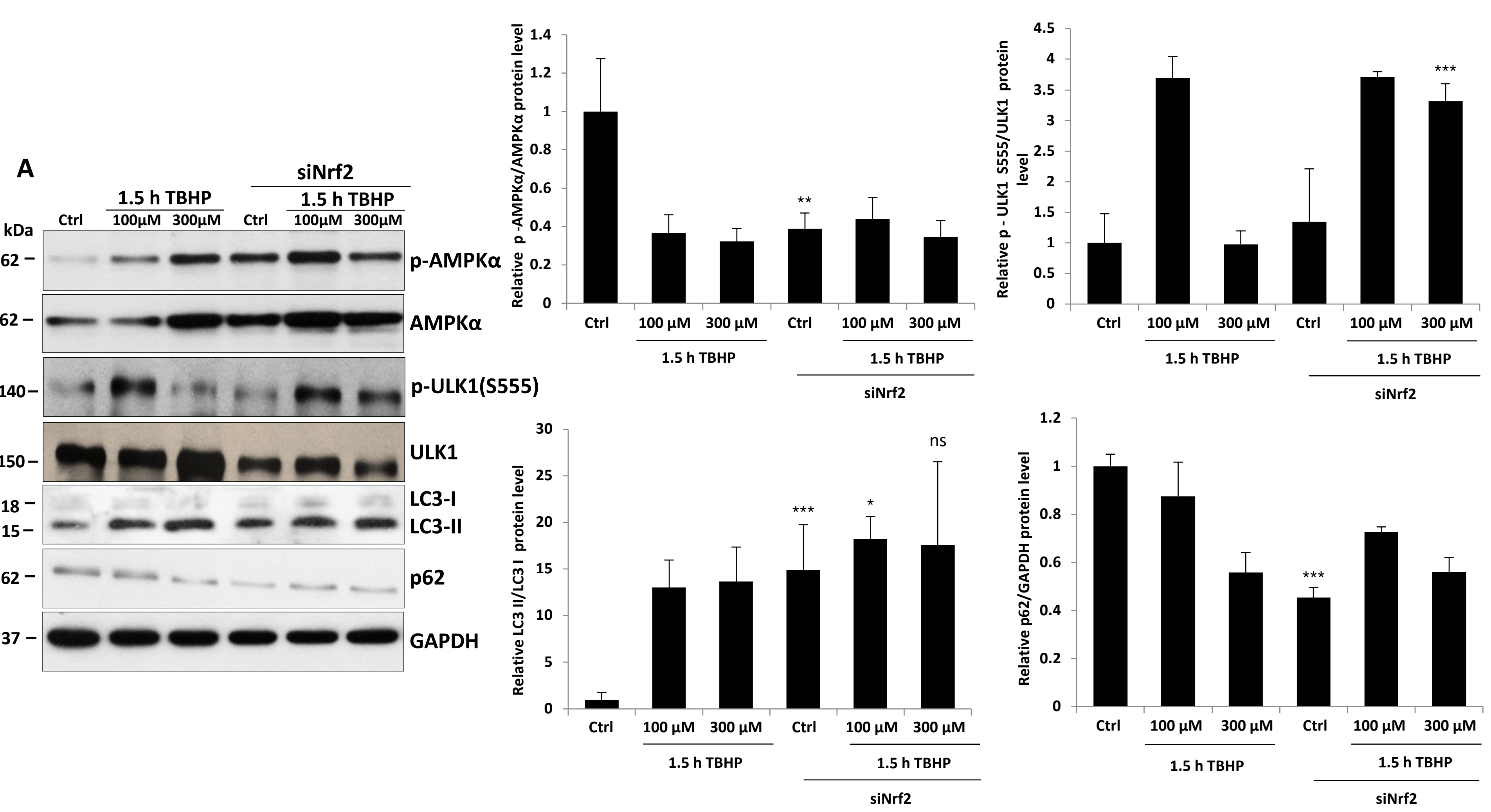

Suppl. Figure 3. Western blot analysis for AMPKα, ULK1, LC3, and other autophagy–related proteins in HEK 293T cell line. (A) Dose-dependency of autophagy down-regulation by NRF2. Western blot results of cells treated with 100 or 300 μM TBHP for 1.5 hours. In half of the samples *NRF2* gene expression was depleted by *NRF2* siRNA. The blots were re-probed/normalized using GAPDH as a loading control. The AMPKα and GADPH blots are the same as those appearing in Suppl. Fig. 2B. **(B)** Quantification and statistical analysis of the Western blot assays. Samples were compared to their partner with siNRF2 background. ***: $p<0.005$, **: $p<0.01$, *: $p<0.05$ (Independent two-sample t-tests). Error bars represent ±SEM

Suppl. Figure 4

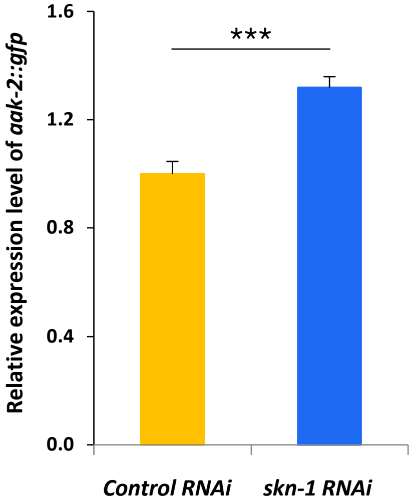

Suppl. Figure 4. SKN-1/NRF2 downregulates *aak-2/AMPK* expression upon oxidative stress in *C. elegans.* Expression of *aak-2::gfp* transgene was increased when *skn-1* gene expression was silenced by *skn-1 RNAi.* ***: $p<0.005$, (Independent two-sample t-tests). Error bars represent ±SEM.

Suppl. Figure 5

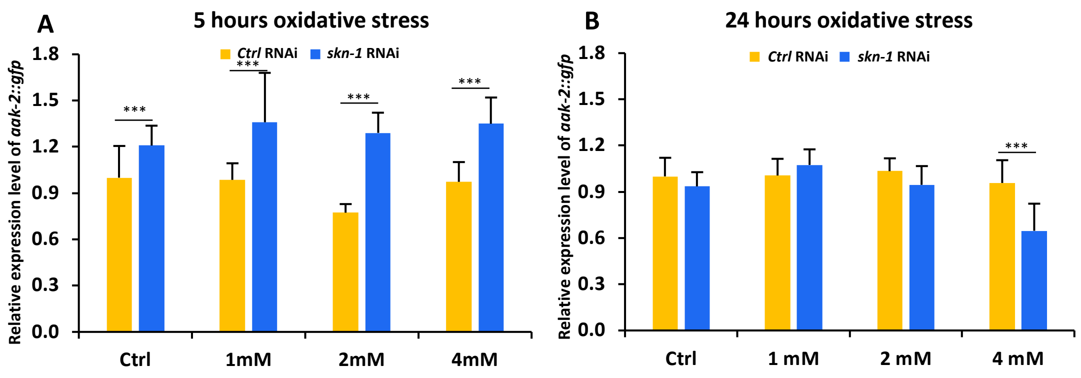

Suppl. Figure 5. Dose dependence of the SKN-1/NRF2 downregulation on *aak-2/AMPK* expression upon oxidative stress in *C. elegans.* Expression of *aak-2::gfp* transgene was increased when *skn-1* gene expression was silenced by *skn-1 RNAi.* ***: $p<0.005$, (Independent two-sample t-tests). Error bars represent ±SEM.
