# Supplementary material for: Suppression of AMPK/aak-2 by NRF2/SKN-1 down-regulates autophagy during prolonged oxidative stress
Source: FASEB J. 2018 Oct 2;33(2):2372–87. doi: 10.1096/fj.201800565RR (PMC6338645; doi:10.1096/fj.201800565RR)
Supplement: Supplementary file 2 [file fj.201800565RR.st1.docx]

Supplementary Tab. 1 Statistical data of Figure 2B and C, Statistics for Western-blot data

| **Condition** | **Duration of 100 µM TBHP treatment** | **Mean relative AMPKα protein density (normalised for GAPDH)** | **±SEM** | **Independent t-test p value vs. control** |
| --- | --- | --- | --- | --- |
| **Control** | **Control** | 1.00 | 0.00 | control |
|  | 0.5 h | 1.77 | 0.04 | vs. control p<0.005 |
|  | 1 h | 1.63 | 0.06 | vs. control p<0.005 |
|  | 2 h | 1.55 | 0.04 | vs. control p<0.005 |
|  | 3 h | 1.18 | 0.04 | vs. control p<0.005 |
|  | 4 h | 1.17 | 0.04 | vs. control p<0.005 |
| **siNRF2** | **Control** | 1.74 | 0.06 | vs. control p<0.005 |
|  | 0.5 h | 1.71 | 0.05 | vs. control 0.5 h TBHP p=0.1892  vs siNRF2 control p=0.2267 |
|  | 1 h | 1.72 | 0.03 | vs. control 1 h TBHP p=0.0828  vs siNRF2 control p=0.2175 |
|  | 2 h | 1.79 | 0.06 | vs. control 2 h TBHP p<0.005  vs siNRF2 control p<0.005 |
|  | 3 h | 1.88 | 0.02 | vs. control 3 h TBHP p<0.005  vs siNRF2 control p<0.005 |
|  | 4 h | 1.89 | 0.04 | vs. control 4 h TBHP p<0.005  vs siNRF2 control p<0.005 |

| **Condition** | **Duration of 100 µM TBHP treatment** | **Mean relative NQO1 protein density (normalised for GAPDH)** | **±SEM** | **Independent t-test p value vs. control** |
| --- | --- | --- | --- | --- |
| **Control** | **Control** | 1.00 | 0.00 | control |
|  | 0.5 h | 1.48 | 0.21 | vs. control p=0.0060 |
|  | 1 h | 1.45 | 0.20 | vs. control p=0.0078 |
|  | 2 h | 1.61 | 0.24 | vs. control p<0.005 |
|  | 3 h | 1.70 | 0.27 | vs. control p<0.005 |
|  | 4 h | 1.53 | 0.26 | vs. control p<0.005 |
| **siNRF2** | **Control** | 0.56 | 0.09 | vs. control p<0.005 |
|  | 0.5 h | 0.60 | 0.10 | vs. control 0.5 h TBHP p<0.005  vs siNRF2 control p=0.0182 |
|  | 1 h | 0.59 | 0.10 | vs. control 1 h TBHP p<0.005  vs siNRF2 control p<0.05 |
|  | 2 h | 0.46 | 0.09 | vs. control 2 h TBHP p<0.005  vs siNRF2 control p<0.005 |
|  | 3 h | 0.60 | 0.10 | vs. control 3 h TBHP p<0.005  vs siNRF2 control p<0.01 |
|  | 4 h | 0.48 | 0.07 | vs. control 4 h TBHP p<0.005  vs siNRF2 control p<0.05 |

| **Condition** | **Duration of 100 µM TBHP treatment** | **Mean relative NRF2 protein density (normalised for GAPDH)** | **±SEM** | **Independent t-test p value vs. control** |
| --- | --- | --- | --- | --- |
| **Control** | **Control** | 1.00 | 0.00 | control |
|  | 0.5 h | 1.21 | 0.09 | vs. control p<0.05 |
|  | 1 h | 1.58 | 0.02 | vs. control p<0.005 |
|  | 2 h | 1.68 | 0.03 | vs. control p<0.005 |
|  | 3 h | 1.82 | 0.03 | vs. control p<0.005 |
|  | 4 h | 1.60 | 0.02 | vs. control p<0.005 |
| **siNRF2** | **Control** | 0.25 | 0.05 | vs. control p<0.005 |
|  | 0.5 h | 0.55 | 0.02 | vs. control 0.5 h TBHP p<0.005  vs siNRF2 control p<0.005 |
|  | 1 h | 0.53 | 0.03 | vs. control 1 h TBHP p<0.005  vs siNRF2 control p<0.005 |
|  | 2 h | 0.57 | 0.03 | vs. control 2 h TBHP p<0.005  vs siNRF2 control p<0.005 |
|  | 3 h | 0.57 | 0.02 | vs. control 3 h TBHP p<0.005  vs siNRF2 control p<0.005 |
|  | 4 h | 0.56 | 0.02 | vs. control 4 h TBHP p<0.005  vs siNRF2 control p<0.005 |

| **Condition** | **Duration of 100 µM TBHP treatment** | **Mean relative HO-1 protein density (normalised for GAPDH)** | **±SEM** | **Independent t-test p value vs. control** |
| --- | --- | --- | --- | --- |
| **Control** | **Control** | 1.00 | 0.00 | control |
|  | 0.5 h | 1.19 | 0.05 | vs. control p<0.005 |
|  | 1 h | 1.25 | 0.06 | vs. control p<0.005 |
|  | 2 h | 1.38 | 0.03 | vs. control p<0.005 |
|  | 3 h | 1.55 | 0.08 | vs. control p<0.005 |
|  | 4 h | 1.13 | 0.05 | vs. control p<0.05 |
| **siNRF2** | **Control** | 0.32 | 0.01 | vs. control p<0.005 |
|  | 0.5 h | 0.59 | 0.03 | vs. control 0.5 h TBHP p<0.005  vs siNRF2 control p<0.005 |
|  | 1 h | 0.66 | 0.04 | vs. control 1 h TBHP p<0.005  vs siNRF2 control p<0.005 |
|  | 2 h | 0.59 | 0.03 | vs. control 2 h TBHP p<0.005  vs siNRF2 control p<0.005 |
|  | 3 h | 0.48 | 0.03 | vs. control 3 h TBHP p<0.005  vs siNRF2 control p<0.005 |
|  | 4 h | 0.35 | 0.03 | vs. control 4 h TBHP p<0.005  vs siNRF2 control p=0.1851 |

Supplementary Tab. 2 Statistical data of Figure S5: Statistics for the relative expression intensity of *aak-2::gfp* reporters (5 and 24h oxidative stress)

| 5 hours oxidative stress | | | | | |
| --- | --- | --- | --- | --- | --- |
| **Condition** | **Final concentration of TBHP treatment** | **Mean of relative expression level** | **±SEM** | **Independent t-test p value vs. Control** | **Number of worms** |
| ***Ctrl RNAi*** | **Control** | 1.00 | 0.04 | control | 28 |
|  | 1 mM | 0.99 | 0.03 | p=0.784 | 19 |
|  | 2 mM | 0.77 | 0.01 | p<0.000 | 23 |
|  | 4 mM | 1.01 | 0.03 | p=0.905 | 14 |
| ***skn-1 RNAi*** | **Control** | 1.21 | 0.02 | p<0.000 | 28 |
|  | 1 mM | 1.36 | 0.74 | vs. *control RNAi* p< 000  vs. *skn-1 RNAi* p<0.05 | 19 |
|  | 2 mM | 1.29 | 0.03 | vs. *control RNAi* p< 000  vs. *skn-1 RNAi* p<0.05 | 17 |
|  | 4 mM | 1.35 | 0.03 | vs. *control RNAi* p< 000  vs. *skn-1 RNAi* p<0.005 | 23 |

| 24 hours oxidative stress | | | | | |
| --- | --- | --- | --- | --- | --- |
| **Condition** | **Final concentration of TBHP treatment** | **Mean of relative expression level** | **±SEM** | **Independent t-test p value vs. Control** | **Number of worms** |
| ***Ctrl RNAi*** | **Control** | 1.00 | 0.04 | control | 16 |
|  | 1 mM | 1.01 | 0.02 | p=0.893 | 15 |
|  | 2 mM | 1.03 | 0.01 | p=0.357 | 16 |
|  | 4 mM | 0.96 | 0.03 | p=0.371 | 17 |
| ***skn-1 RNAi*** | **Control** | 0.94 | 0.02 | p=0.106 | 15 |
|  | 1 mM | 1.07 | 0.02 | vs. *control RNAi* p=0.071  *skn-1 RNAi* p<0.000 | 18 |
|  | 2 mM | 0.95 | 0.03 | vs. *control RNAi* p<0.05  vs. *skn-1 RNAi* p=<0.796 | 18 |
|  | 4 mM | 0.65 | 0.04 | vs. *control RNAi* p< 0.000  vs. *skn-1 RNAi* p<0.000 | 15 |

Supplementary Tab. 3 Statistical data of Figure 6E: Statistics for the paralysis assay of worms in oxidative stress (10 mM TBHP, 7 hours; with or without *skn-1* depletion by *skn-1* RNAi)

| **7 hours TBHP treatment (10mM)** | ***N2***  ***Control RNAi*** | ***N2***  ***skn-1 RNAi*** | ***aak-2(gt33)***  ***Control RNAi*** | ***aak-2(gt33) skn-1 RNAi*** | ***skn-1(lax120) Control RNAi*** | ***skn-1(lax120) skn-1 RNAi*** | **Number of animals** |
| --- | --- | --- | --- | --- | --- | --- | --- |
| ***N2 Control RNAi*** | - | p=0.678 | p=0.033 | - | p=0.210 | - | 47 |
| ***N2 skn-1 RNAi*** | p=0.678 | - | - | p=0.059 | - | p=0.093 | 41 |
| ***aak-2(gt33) Control RNAi*** | p=0.033 | - | - | p=0.198 | p=0.393 | - | 67 |
| ***aak-2(gt33) skn-1 RNAi*** | - | p=0.059 | p=0.198 | - | - | p=0.527 | 90 |
| ***skn-1(lax120) Control RNAi*** | p=0.210 | - | p=0.393 | - | - | p=0.106 | 48 |
| ***skn-1(lax120) skn-1 RNAi*** | - | p=0.093 | - | p=0.527 | p=0.106 | - | 52 |

Supplementary Tab. 4 Statistical data of Figure 7A and B

| **Strain** | **Condition** | **Relative ratio of GFP::LGG-1 positive area in the cells** | **±SEM** | **Independent t-test p value vs. control** | **Number of animals** |
| --- | --- | --- | --- | --- | --- |
| **N2** | Control (HT115) | 2.539 | 0.530 | control | 36 |
|  | Control (HT115),  1mM TBHP | 0.540 | 0.302 | vs.control p<0.0001 | 20 |
|  | *skn-1(RNAi)* | 1.791 | 0.421 | vs.control p=0.3308 | 9 |
|  | *skn-1(RNAi),*1 mM TBHP | 0.420 | 0.180 | vs.N2 HT115, 1mM TBHP p<0.0001  vs N2 *skn-1 (RNAi)* p<0.0001 | 7 |
| ***aak-2(gt33)*** | Control (HT115) | 2.350 | 0.262 | vs.N2 HT115 p=0.000989 | 8 |
|  | Control (HT115),  1mM TBHP | 1.415 | 0.241 | vs.N2 HT115, 1mM TBHP p<0.0001  vs. *aak-2(gt33),* HT115 p<0.0001 | 13 |
|  | *skn-1(RNAi)* | 1.209 | 0.217 | vs*. aak-2(gt33)* ,HT115 p<0.0001 | 10 |
|  | *skn-1(RNAi),* 1 mM TBHP | 0.700 | 0.176 | vs. *aak-2(gt33)* HT115, 1mM TBHP p<0.0001  vs. *aak-2(gt33*), *skn-1 (RNAi)* p<0.0001 | 11 |

Supplementary Tab. 5 Statistical data of Figure 7C, Statistics for the paralysis assay of worms in oxidative stress (7 hours, 10 mM TBHP)

| **7 hours TBHP treatment** | **N2 Control RNAi**  **10 mM TBHP** | **N2 *skn-1 RNAi***  **10 mM TBHP** | ***atg-11(tm2508)* Control RNAi**  **10 mM TBHP** | ***atg-11(tm2508)***  ***skn-1 RNAi***  **10 mM TBHP** | **Number of animals** |
| --- | --- | --- | --- | --- | --- |
| **N2 Control RNAi 10 mM TBHP** | - | p=0.678 | p=0.000 | p=0.019 | 47 |
| **N2 *skn-1 RNAi* 10 mM TBHP** | p=0.678 | - | - | - | 41 |
| ***atg-11(tm2508)* Control RNAi**  **10 mM TBHP** | p=0.000 | - | - | p=0.044 | 61 |
| ***atg-11(tm2508*) *skn-1 RNAi***  **10 mM TBHP** | p=0.019 | - | p=0.044 | - | 43 |
